# Successful long-term extracorporeal membrane oxygenation for invasive pulmonary aspergillosis: a case report

**DOI:** 10.1186/s13256-017-1381-5

**Published:** 2017-08-11

**Authors:** Hiroyuki Tanaka, Kei Nishiyama, Nobuaki Shime

**Affiliations:** 1 Department of Emergency and Critical Care Medicine, National Hospital Organization, Kyoto Medical Center, 1-1 Fukakusa, Mukaihata-cho, Fushimi-ku, 612-8555 Kyoto Japan; 2 Department of Emergency and Critical Care Medicine, Hiroshima University, Hiroshima, Japan

**Keywords:** Respiratory failure, Extracorporeal membrane oxygenation (ECMO)

## Abstract

**Background:**

Extracorporeal membrane oxygenation is an established life-saving procedure for severe acute respiratory failure due to various causes. In general, the duration of extracorporeal membrane oxygenation ranges from 1 to 2 weeks, with withdrawal recommended if no improvement is noted. We report a successful case of long-term extracorporeal membrane oxygenation management for respiratory failure due to invasive pulmonary *Aspergillus* infection.

**Case presentation:**

A 64-year-old Asian man with no previous underlying medical conditions was transferred to our hospital for fever and dyspnea. On admission, he presented with bilateral diffuse infiltration shadow on X-ray and chest computed tomography readings, and severe hypoxemia with a partial pressure of oxygen in arterial blood/fraction of inspired oxygen ratio of 55. He was intubated and underwent mechanical ventilation. A bronchial-alveolar lavage was performed prior to administration of antibiotics, and as the bacterial culture was positive for *Aspergillus fumigatus*, antifungal treatment was then initiated. His respiratory status deteriorated on the 11th admission day, with no improvement on any mechanical ventilator settings. Venous-venous extracorporeal membrane oxygenation was introduced. Extracorporeal membrane oxygenation was used for an extended period of time, with respiratory improvement delayed until the 39th admission day. Extracorporeal membrane oxygenation discontinuation was possible on the 44th day, and he was removed from the ventilator on the 64th day.

**Conclusions:**

Long-term extracorporeal membrane oxygenation might be considered if the primary causes of respiratory failure necessitating extracorporeal membrane oxygenation can be expected to be resolved, such as in the case of effective antimicrobial therapy for a definite pathogen. Our case indicates that extracorporeal membrane oxygenation can be used during treatment of respiratory failure due to invasive aspergillosis for the recommended treatment duration of 4 to 8 weeks.

## Background

**Fig. 1:**
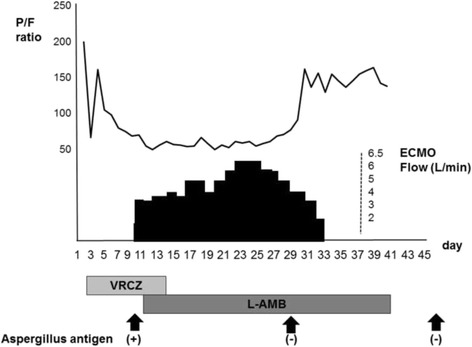
Clinical course of the patient. Trends in the partial pressure of oxygen in arterial blood/fraction of inspired oxygen ratio, extracorporeal membrane oxygenation flow, and antifungal treatment history are shown. *ECMO* extracorporeal membrane oxygenation, *L-AMB* liposomal amphotericin B, *P/F ratio* partial pressure of oxygen in arterial blood/fraction of inspired oxygen ratio, *VRCZ* voriconazole

Extracorporeal membrane oxygenation (ECMO) therapy has shown promising results for patients with severe respiratory failure unresponsive to conventional mechanical ventilation [1]. However, there have been a number of conflicting guidelines concerning this therapy, in particular, regarding the duration of therapy. The Extracorporeal Lung Support Organization (ELSO) guidelines suggest a treatment period of 2 weeks and withdrawal if there is no improvement [2]. This period, however, might be varied according to the primary cause of respiratory failure and, in some cases, prolonged application might be associated with improved outcome. We report a case of severe respiratory failure caused by invasive aspergillosis in a patient who was taken off ECMO therapy after 33 days (44 days from admission). A brief clinical course of the patient is shown here (Fig. 1).

## Case presentation

**Fig. 2:**
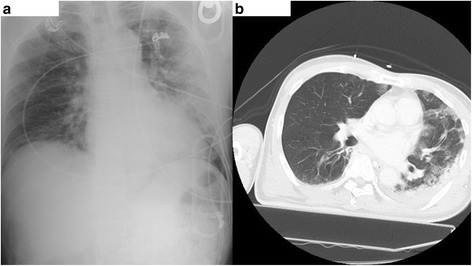
Imaging studies on admission. **a** Chest X-ray. *Left* dominant bilateral infiltration shadow is noted. **b** Computed tomography scan. *Left* dominant bilateral infiltration shadow with *small* pleural effusion is noted

A 64-year-old Asian man was transferred to our hospital for fever and dyspnea. He had no previous underlying medical conditions, allergies, or medications. He smoked approximately 1 pack of cigarettes per day and drank occasionally. He had no particular family history issues and had an occupational history of working in a dusty environment, but with no exposure to asbestos. On admission, he was severely dyspneic with a respiratory rate of 34 breaths/minute and a pulse oximetry reading of 93% under 15 L/minute oxygen administration. His blood pressure was 101/59 mmHg, his heart rate was 109 beats/minute, and he was feverish with a body temperature of 39.7 °C (103.5 °F). Auscultation revealed bilateral crackles in all lung fields. Blood tests showed mildly elevated liver enzymes, decreased renal functions, and elevated C-reactive proteins with no elevation of white blood cells. X-ray imaging showed bilateral diffuse infiltration shadows (Fig. 2). Arterial blood gas analysis showed type I respiratory failure with a partial pressure of oxygen in arterial blood/fraction of inspired oxygen (PaO_2_/F_I_O_2_) ratio of 55. The results of the rapid diagnostic testing for influenza was positive for influenza A. Computed tomography (CT) scan imaging showed bilateral infiltrates with a small amount of bilateral pleural effusion (Fig. 2). His Acute Physiology and Chronic Health Evaluation (APACHE) II score on admission was 23 points, with a predicted mortality rate of 47%.

Since both he and his family were not in favor of mechanical ventilation, oxygen therapy alone was initiated for respiratory failure. However, his respiratory condition deteriorated on the second admission day and he complained of severe dyspnea. Informed consent was obtained for intensive therapy, and he was intubated and underwent mechanical ventilation on the same day.

**Fig. 3:**
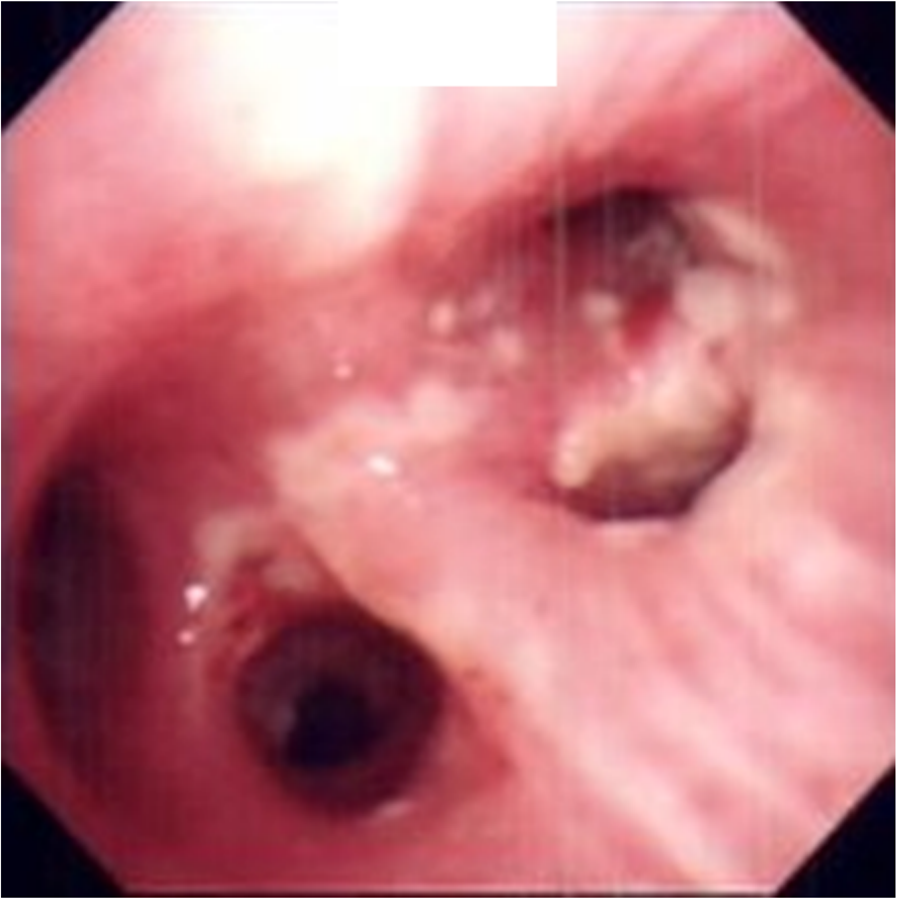
Bronchofiberscopy images. White exudates are noted in the orifice of *right* upper lobe branch of bronchus

Bronchoalveolar lavage was performed prior to administration of antibiotics. White exudates were noted in the orifice of right upper lobe branch of bronchus (Fig. 3). Empirical therapy with ceftriaxone and azithromycin was initiated. A bacterial culture was positive for *Aspergillus*. Antifungal treatment with intravenously administered voriconazole and liposomal amphotericin B was initiated. The test result for *Aspergillus* antigen was positive (4.0+). His respiratory status remained stable, but suddenly deteriorated on the 11th admission day to a PaO_2_/F_I_O_2_ ratio of less than 50, even under high positive end-expiratory pressure (PEEP) settings of 15 mmH_2_0 and an F_I_O_2_ setting of 1.0. His respiratory status did not improve in response to any mechanical ventilator settings or rescue therapies such as prone positioning or recruitment maneuvers. Informed consent was obtained for further intensive therapy, and venous-venous ECMO was introduced. A 16-French return catheter was placed in his right internal jugular vein with the distal tip in his superior vena cava, and a 20-French drainage catheter was placed in his right femoral vein with the distal tip in his inferior vena cava at thoracic level 10 (T10).

**Fig. 4:**
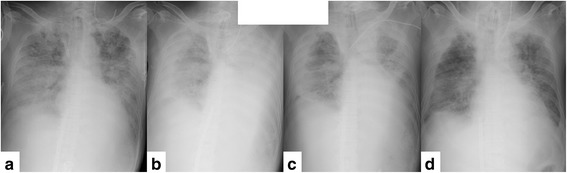
Trends in X-ray imaging. **a** Day 11. **b** Day 22. **c** Day 30. **d** Day 34. Note the drastic improvement on day 30

The initial setting for ECMO was a flow of 3.5 L/minute, with an F_I_O_2_ setting of 0.8. There was no major improvement in his respiratory status between the ninth and 16th days – that is, seventh and 14th day of ECMO introduction, respectively – with PaO_2_/F_I_O_2_ ratios measured by right radial arterial blood gas analysis falling between 50 and 60. While discontinuation of ECMO therapy was discussed among caregivers and families, his PaO_2_/F_I_O_2_ ratio improved to 85 and a chest X-ray showed decreasing infiltration shadows on the 28th day of ECMO. The settings were adjusted such that he was cautiously weaned off ECMO, and discontinuation was possible on the 33rd day of ECMO (44th admission day); he was free of ventilator support on the 66th day. He was transferred to a general ward on the 103rd day and to a rehabilitation facility on the 176th day. Trends in X-ray imaging are shown here (Fig. 4).

The test result for *Aspergillus* antigen was confirmed negative on the 28th admission day and reconfirmed on the 53rd day. The treatment with antifungal agents was as follows: voriconazole loading with 400 mg on the second admission day and continued with 200 mg every 12 hours from the third day to the 16th day, liposomal amphotericin B at 2.5 mg/kg for the tenth day to the 11th day, and an escalated dose of liposomal amphotericin B of 5.0 mg/kg from the 12th day to the 45th day. Although there was a slight elevation in liver enzymes, there were no major adverse effects due to administration of these drugs. There was no further administration of antifungal agents after the 45th day. At the point of transfer to the rehabilitation facility, there were no other medical prescriptions. After discharge, follow-up with diagnostic imaging and laboratory testing was performed every 3 months for a period of 1 year, with no major changes in laboratory test results and X-ray imaging.

## Discussion

To the best of our knowledge, this is the first report demonstrating successful long-term ECMO therapy for acute respiratory failure due to invasive pulmonary aspergillosis.

The conventional ventilatory support versus ECMO for severe adult respiratory failure (CESAR) trial in 2009 showed that ECMO therapy can improve patient outcome in terms of survival rate for severe respiratory failure [1], and a number of patients have received ECMO therapy for respiratory failure since then. Further studies have shown that prolonged ECMO is associated with a 45% hospital survival rate [3]. In addition, there have been case reports of successful ECMO therapy in the elderly population, with a survival rate of 41% [4].

Conversely, ELSO guidelines suggest a standard period of 2 weeks for working duration, and withdrawal if there is no improvement, given futility and cost-efficacy concerns [2]. The number of complications – including infection and coagulopathy [5] – associated with ECMO increases during persistent treatment, which may prompt clinical decisions to terminate treatment after a prescribed period of time. However, this therapy may be extended if the primary causes of respiratory failure necessitating ECMO can be expected to resolve. Acute respiratory infection with a defined pathogen(s) as a sole cause of the respiratory failure can be a candidate for extended ECMO treatment. Data on successful outcomes in the 2009 H1N1 influenza outbreak support the possibility of applying long-term ECMO treatment to acute respiratory failure caused by a known organism when accompanied by appropriate chemotherapy [6]. The recommended treatment duration for invasive aspergillosis ranges from at least 4 to 8 weeks [7], indicating a possibility for applying long-term ECMO to this specific disease. Our patient, in fact, could be treated by effective chemotherapy for >4 weeks.

Complications such as coagulopathies – including bleeding tendency and thrombi formation – and infection may lead to shortening or termination of ECMO therapy [3]. Our experience shows that the duration of ECMO therapy may be prolonged by successfully managing these complications. In addition, successful definitive therapy for the primary cause of respiratory failure is another key to prolonged ECMO treatment.

## Conclusions

We encountered a case of 33-day ECMO management for severe respiratory failure due to invasive pulmonary aspergillosis. Our experience shows that ECMO may be used for a longer period than is conventional if the primary cause of respiratory failure can be resolved with specific treatment.

## Abbreviations

APACHE: Acute Physiology and Chronic Health Evaluation
CT: Computed tomography
ECMO: Extracorporeal membrane oxygenation
ELSO: Extracorporeal Lung Support Organization
F_I_O_2_: Fraction of inspired oxygen
PaO_2_: Partial pressure of oxygen in arterial blood, PEEP, Positive end-expiratory pressure
T10: Thoracic level 10
